# 

**Published:** 1906-01

**Authors:** 


### CONTENTS.

ORIGINAL COMMUNICATIONS—

Conservative Gynecology. By W. A. Verdier, M.D., Atlanta, Ga .......... 649
A Successful Cesarean Section in Country Practice. By S. A. Reynolds, M.D., Vashti, Va .......... 657
Prevention of Renal Diseases. By Wm. T. Jones, M.D., Atlanta, Ga .......... 661
Corneal Abscission—A Report of Forty-one Operations. By J. M. Crawford, M.D., Atlanta, Ga .......... 663

EDITORIAL NOTES AND COMMENTS—

More About Quacks .......... 675
Preventive Medicine .......... 678

NEWS AND NOTES .......... 680

BOOK REVIEWS—

Progressive Medicine. (Hare) .......... 684
Cleft Palate and Hare Lip. (Lane) .......... 684
LeFevre's Diagnosis. (LeFevre) .......... 685
A Text-Book of Physiology, Normal and Pathological. (Hall) .......... 685
The Surgical Diseases of the Genito-Urinary Tract, Venereal and Sexual Diseases. (Lydston) .......... 686
The Physician's Visiting List for 1906. (Lindsay & Blakiston) .......... 686
Forty Years an Advertising Agent. (Rowell) .......... 686

CONTENTS CONTINUED .......... x

